# Prenatal diagnosis and genetic counseling of a 10p11.23q11.21 duplication associated with normal phenotype

**DOI:** 10.1186/s13039-022-00598-x

**Published:** 2022-06-03

**Authors:** Jieping Song, Wei Jiang, Chengcheng Zhang, Bo Wang

**Affiliations:** Department of Clinical Laboratory, Maternal and Child Health Hospital of Hubei Province, Wuhan, Hubei People’s Republic of China

**Keywords:** Chromosomal microarray analysis (CMA), Noninvasive prenatal testing (NIPT), Chromosomal deletions/duplications, Prenatal diagnosis, Unbalanced chromosomal abnormalities (UBCA)

## Abstract

**Background:**

Copy number variants (CNVs) are an important source of normal and pathogenic genome variations. Unbalanced chromosome abnormalities (UBCA) are either gains or losses or large genomic regions, but the affected person is not or only minimally clinically affected. CNVs and UBCA identified in prenatal cases need careful considerations and correct interpretation if those are harmless or harmful variants from the norm.

**Case presentation:**

A 24-year-old, gravida 1, para 0, woman underwent amniocentesis at 17 weeks of gestation because the noninvasive prenatal testing (NIPT) results revealed a 12.4 Mb duplication from 10p11.2 to 10q11.2. GTG-banding karyotype analysis was performed on cultured amniocytes. Chromosomal microarray analysis (CMA) on uncultured amniocytes was performed.

**Results:**

Chromosomal GTG-banding of the cultured amniocytes revealed a karyotype of 46,XX,dup(10)(p11.2q11.2). CMA detected a 12.5-Mb chromosomal duplication in the region of 10p11.23q11.21 (arr[GRCh37] 10p11.23q11.21(30,345,109_42,826,062) × 3).

**Conclusion:**

The present report enlarges the known UBCA region 10p11.22-10q11.22 to 10p11.23-10q11.22. Also it highlights that an integration of prenatal ultrasound, NIPT, karyotype analysis, CMA and genetic counseling is helpful for the prenatal diagnosis of chromosomal deletions/duplications.

## Introduction

Unbalanced chromosomal abnormalities (UBCA) were reported for euchromatic regions of many human autosomes. Carriers of UBCA are in many cases clinically healthy, and UBCA are often nothing else than cytogenetically visible copy number variants (CNVs) [1].

Noninvasive prenatal testing (NIPT) is widely used in the screening of common fetal chromosome aneuploidy [2]. Conventional karyotyping provides an overview of the entire genome and can identify structural and numerical chromosome abnormalities. Chromosomal microarray analysis (CMA) is a method using array technology to detect chromosome abnormalities spanning less than 5 Mb [3]. Because CMA does not require cell culture, samples which cannot be cultured by conventional karyotyping can be analyzed with CMA, and CMA offers faster testing result. However, conventional karyotyping is limited to detect the rearrangement with a length longer than 5 Mb, which can be detected by CMA [4] and CMA cannot detect balanced translocations, which can be detected by conventional karyotyping [5].

Here we report the prenatal diagnosis and genetic counseling of a novel 10p11.23q11.21 duplication in a Chinese family with normal phenotype using NIPT, chromosomal GTG-banding and CMA.

## Methods

### Patients and samples

In 2018, a 24-year-old, gravida 1, para 0, woman underwent amniocentesis at 17 weeks of gestation because the noninvasive prenatal testing (NIPT) results revealed 12.4 Mb duplication from 10p11.2 to 10q11.2. Her husband was 25-year old. There was no family history of birth defects or genetic diseases. GTG-banding karyotype analysis was performed on cultured amniocytes and parental blood samples. CMA on uncultured amniocytes was performed using the Affymetrix CytoScan 750 K chip, which includes 550 k non-polymorphic markers and 200 k SNP markers.

## Results

**Fig. 1:**
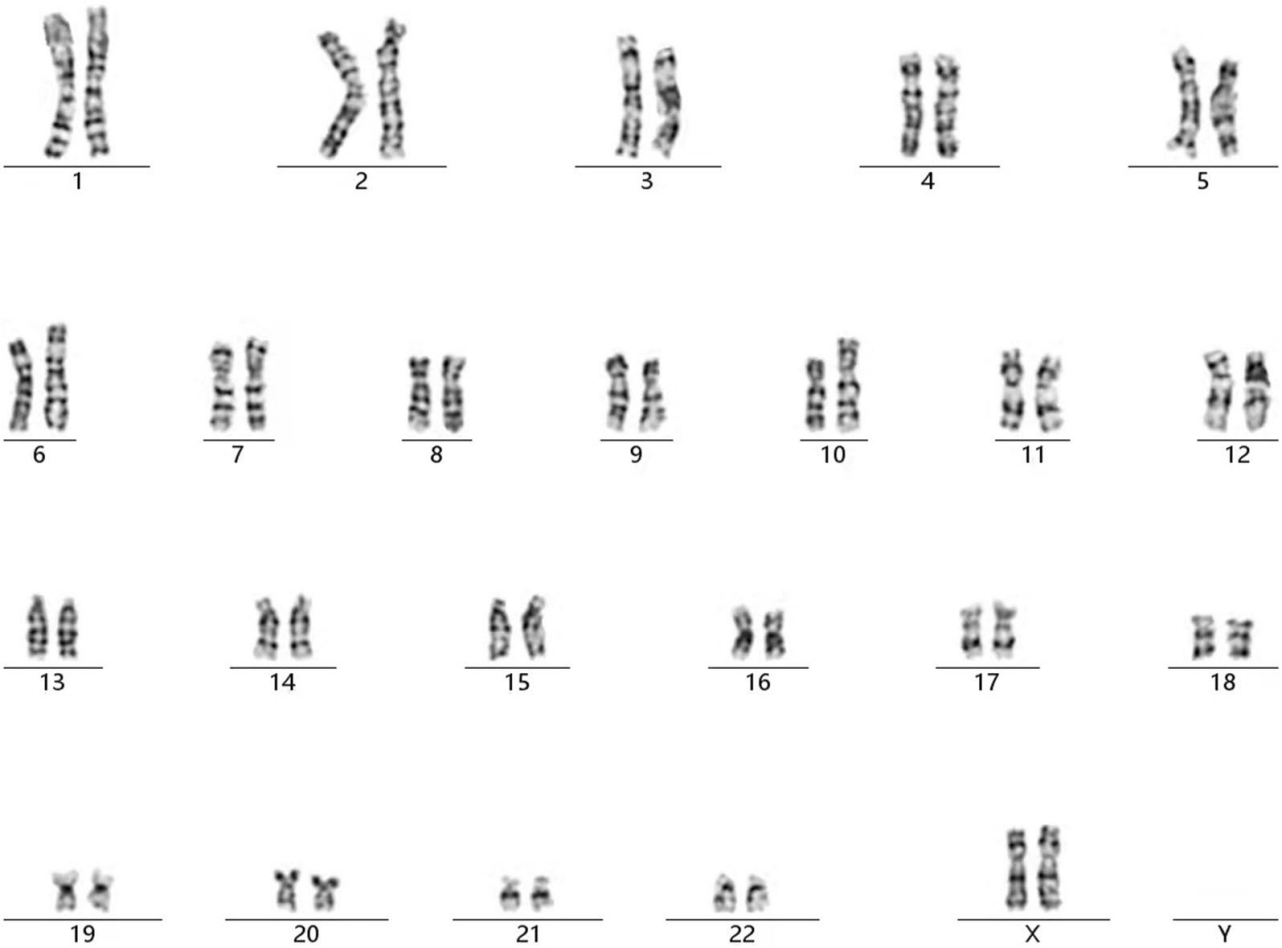
The karyotype of patient with dup(10)(p11.2q11.2)

**Fig. 2:**
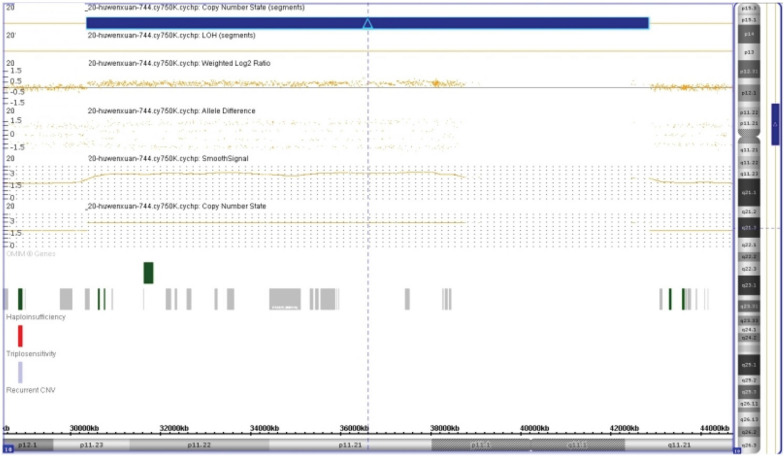
CMA detected a 12.5-Mb chromosomal duplication in the region of 10p11.23q11.21 (arr[GRCh37]10p11.23q11.21(30,345,109_42,826,062) × 3)

Chromosomal GTG-banding revealed a karyotype of 46,XX,dup(10)(p11.2q11.2) (Fig. 1). CMA detected a 12.5-Mb chromosomal duplication in the region of 10p11.23q11.21, which is to be reported according to International System of Cytogenomic Nomenclature 2020 (ISCN 2020) [6] as arr[GRCh37] 10p11.23q11.21(30,345,109_42,826,062) × 3 (Fig. 2). Then we performed both CMA and chromosomal GTG-banding using the samples from the parents' peripheral blood. Their karyotypes and CMA were normal. Ultrasound examination showed no dysmorphisms or intrauterine growth restriction (IUGR) in the fetus. At 24 weeks of gestation, this fetus had an estimated fetal weight of 670 g, an abdominal circumference of 19.7 cm, a head circumference of 21.9 cm, a femur length of 4.3 cm and a fetal heart rate of 145 bpm [7]. After genetic counseling, the parents decided to continue the pregnancy.

At 40 weeks of gestation, the expectant mother gave birth vaginally to a female baby. The baby's growth parameters at birth were in the normal ranges. Apgar scores were 9/10/10. The baby received a complete physical examination and the results were normal. At 36-month checkup, the baby was developing normally (Intelligence Quotient, IQ = 110).

## Discussion

According to the literature [1, 8–12] yet only several cases/ families with partial trisomies of chromosome 10 within the pericentromeric region are reported, which did not show any or minimal clinical signs. Different from trisomy 10p syndrome, the pericentromeric region of chromosome 10 is proposed [11] to be a triplo-insensitive pericentromeric region.

In this study, the chromosomal duplication of 10p11.23q11.21 contains 25 genes, and these 25 genes are all triplo-insensitive genes. We report the partly dup(10) with a long-term postnatal (3 years) follow-up. To the best of our knowledge, this is the first report of an UBCA in the pericentromeric region of chromosome 10 that is not correlated with any clinical consequences, thus enlarging the yet known region 10p11.22-10q11.22 to 10p11.23-10q11.22.

Predicting the phenotypic outcome of prenatally diagnosed de novo partial dup(10) remains challenging. Important efforts have been devoted to define chromosome non-critical pericentromeric regions that tolerate duplication without phenotypic effects, a key issue in genetic counseling [8]. Unfortunately, most defined non-critical regions remain speculative at present, because available information is scarce.

Partial trisomies of chromosome 10 in the pericentromeric region were identified prenatally in several cases. A maximum of three copies of the region from 10p11.22 to 10q11.22 was observed in all cases without apparent clinical abnormalities. The imbalances were either caused by a direct duplication in one familial case or by de novo small supernumerary marker chromosomes (sSMC) [1].

On the other hand, patients with partial tetrasomy of chromosome 10 or partial trisomies of 10p (the trisomy 10p syndrome) have malformation of various organs, hypotonia, developmental delay, skeletal abnormalities and seizures [9].

During pregnancy, there were no dysmorphisms or IUGR in the fetus. At the 3-year follow-up, the baby did not have an abnormal phenotype and exhibited no evidence of developmental delay. This observation provided credence to the concept that trisomies of 10p11.23q11.21 may not contribute to abnormal phenotype. However, further study is needed to understand the expression of these 25 genes in triplicate condition and its pathogenic affect. We plan to follow this patient in order to monitor her development.

NIPT is a very efficient and accurate method for the detection of chromosome aneuploidy, especially for chromosome 13, 18 and 21. Recently, further expansion of NIPT through deeper sequencing has focused on additional screening for microdeletion and microduplications, which had also notable screening results [13].

CMA is superior to standard karyotype in detection of chromosomal microdeletion/microduplication [14]. Therefore, CMA is recommended as an additional prenatal screening item while conventional prenatal tests including blood test, ultrasonography examination and invasive prenatal diagnosis revealed abnormal findings of fetus [15].

## Conclusions

Combination of prenatal ultrasound, karyotype analysis, NIPT, CMA and genetic counseling is helpful for the prenatal diagnosis of chromosomal microdeletions/microduplications.

## Data Availability

All relevant data and material is included in this publication.

## Abbreviations

UBCA: Unbalanced chromosome abnormalities
CNVs: Copy number variants
CMA: Chromosomal microarray analysis
NIPT: Noninvasive prenatal testing
IUGRs: Intrauterine growth restrictions
